# A novel computational framework for the estimation of internal musculoskeletal loading and muscle adaptation in hypogravity

**DOI:** 10.3389/fphys.2024.1329765

**Published:** 2024-02-07

**Authors:** James Cowburn, Gil Serrancolí, Gaspare Pavei, Alberto Minetti, Aki Salo, Steffi Colyer, Dario Cazzola

**Affiliations:** ^1^ Department for Health, University of Bath, Bath, United Kingdom; ^2^ Centre for the Analysis of Motion, Entertainment Research and Applications, University of Bath, Bath, United Kingdom; ^3^ Department of Mechanical Engineering, Universitat Politècnica de Catalunya, Barcelona, Spain; ^4^ Department of Pathophysiology and Transplantation, University of Milan, Milan, Italy

**Keywords:** plyometric hopping, musculoskeletal modelling, musculoskeletal load, muscle adaptation model, body weight support, tracking simulation, direct collocation

## Abstract

**Introduction:** Spaceflight is associated with substantial and variable musculoskeletal (MSK) adaptations. Characterisation of muscle and joint loading profiles can provide key information to better align exercise prescription to astronaut MSK adaptations upon return-to-Earth. A case-study is presented of single-leg hopping in hypogravity to demonstrate the additional benefit computational MSK modelling has when estimating lower-limb MSK loading.

**Methods:** A single male participant performed single-leg vertical hopping whilst attached to a body weight support system to replicate five gravity conditions (0.17, 0.25, 0.37, 0.50, 1 g). Experimental joint kinematics, joint kinetics and ground reaction forces were tracked in a data-tracking direct collocation simulation framework. Ground reaction forces, sagittal plane hip, knee and ankle net joint moments, quadriceps muscle forces (Rectus Femoris and three Vasti muscles), and hip, knee and ankle joint reaction forces were extracted for analysis. Estimated quadriceps muscle forces were input into a muscle adaptation model to predict a meaningful increase in muscle cross-sectional area, defined in (DeFreitas et al., 2011).

**Results:** Two distinct strategies were observed to cope with the increase in ground reaction forces as gravity increased. Hypogravity was associated with an ankle dominant strategy with increased range of motion and net plantarflexor moment that was not seen at the hip or knee, and the Rectus Femoris being the primary contributor to quadriceps muscle force. At 1 g, all three joints had increased range of motion and net extensor moments relative to 0.50 g, with the Vasti muscles becoming the main muscles contributing to quadriceps muscle force. Additionally, hip joint reaction force did not increase substantially as gravity increased, whereas the other two joints increased monotonically with gravity. The predicted volume of exercise needed to counteract muscle adaptations decreased substantially with gravity. Despite the ankle dominant strategy in hypogravity, the loading on the knee muscles and joint also increased, demonstrating this provided more information about MSK loading.

**Discussion:** This approach, supplemented with muscle-adaptation models, can be used to compare MSK loading between exercises to enhance astronaut exercise prescription.

## 1 Introduction

Spaceflight presents a substantial physiological challenge to the human body. Astronauts can present with substantial muscular adaptations, such as atrophy and reduced strength, power, and endurance, in as little as 7–14 days of spaceflight (Winnard et al., 2019). To complicate matters, substantial inter-individual variability has been reported in the literature (Fernandez-Gonzalo et al., 2021; Scott et al., 2021), both in terms of the magnitude and the nature of the muscular adaptations experienced by astronauts. For example, Widrick et al. (1999) reported a 2-fold variation in responses of muscle contractile properties following 17 days spaceflight. While, Rittweger et al. (2018) showed that astronauts may or may not present with muscle architectural adaptations (i.e., fibre length and pennation angle) alongside muscle atrophy following long-term spaceflight. Despite comprehensive post-spaceflight rehabilitation programmes (Lambrecht et al., 2017) some aspects of the musculoskeletal system may not return to pre-flight condition after 1-year (Kramer et al., 2018). Therefore, the selection of specific exercises to align with the individual adaptations experienced by the astronaut to personalise their rehabilitation is key. This would require characterisation of the loading profiles and the musculoskeletal (MSK) structures being loaded (i.e., individual muscles and joints) during spaceflight relevant exercises.

Musculoskeletal modelling presents a powerful tool that can be used to characterise biomechanical loading. Information about skeletal anatomy and muscle-tendon unit (MTU) physiology, including geometry, contraction dynamics, and neural control, are used to replicate the MSK system to analyse human movement. When combined with optimisation techniques to predict activation patterns it is possible to estimate internal loading variables (e.g., muscle forces and joint reaction forces) that are otherwise not feasible *in vivo*. This information has been used in other clinical contexts to inform rehabilitation of patients. MSK modelling has allowed researchers to identify alternative muscle activation strategies to reduce joint contact forces (DeMers et al., 2014), and to distinguish between clinical populations based on their joint contact loading profiles (e.g., Saxby et al., 2016; Wesseling et al., 2018). Furthermore, Van Rossom and colleagues (2018) quantified the magnitude and location of tibiofemoral and patellofemoral contact forces for common rehabilitation exercises. As they suggested, this type of information can be used to grade exercises according to their biomechanical loading profile, which can be better aligned to the patient’s injury and rehabilitation stage. This is particularly relevant to astronaut exercise prescription, both in terms of selection and timing of exercises, which has previously been driven by expert opinion rather than scientific evidence (Loehr et al., 2015; Lambrecht et al., 2017). As mentioned above, what is apparent is that muscular adaptations persist during spaceflight and rehabilitation post-flight is slow. For some MSK tissue it may not be possible to achieve pre-flight conditioning with current knowledge. Employing MSK modelling within a hypogravity context would better address this knowledge gap by allowing for internal load on the muscles and joints to be estimated. Exercises can then be identified to better align MSK loading with clinical outcomes and an astronaut’s MSK condition. For example, there has been a recent shift in focus towards plyometric jumping exercises as an in-flight countermeasure (Kramer et al., 2017; Kramer et al., 2018; Weber et al., 2019). The proposed benefit of repetitive, short-duration but high-loading associated with plyometric exercise can be compared to other exercises (e.g., walking and running) to understand whether plyometric exercises represent a more efficient method for mitigating against and rehabilitating from MSK adaptations to spaceflight. Demonstrating this added value of MSK modelling is a key step in increasing it is implementation in practice (Killen et al., 2020; Fregly, 2021), and would supplement previous expert knowledge to optimise astronaut exercise prescription.

There is a premium on time-resources during spaceflight due to a wide variety of operational commitments astronauts are required to perform during flight. Currently, on the International Space Station astronauts can spend 2.5 h a day exercising to maintain MSK health, with a combination of resistance and aerobic apparatus available in-flight (Loehr et al., 2015). Little information is available that describes how specific exercises are selected, but recommended programs are regularly altered to accommodate astronaut preference and, under high-loads, comfort of using the machines (Loehr et al., 2015). It is difficult to ascertain what the exact limitations are in current in-flight exercise paradigms, but sub-optimal exercise selection, particularly when substituting exercises to suit astronauts, may be a contributing factor. Given the time resource and the inability to fully mitigate against muscular adaptations, there is still a critical knowledge gap in understanding how exercise and hypogravity exposure can be optimised to preserve astronaut health. Although the internal loading estimated via MSK modelling provides useful information to monitor progressive overload, it is difficult to directly use this knowledge to design a training program (e.g., number of sets and repetitions).

One approach to use the outputs to design in-flight and post-spaceflight exercise programmes is to combine them with mathematical relationships between MSK load and muscular adaptations in response to under-loading, overloading, under-stretch and over-stretch (Zöllner et al., 2012; Wisdom et al., 2015; Zhou et al., 2017). Estimating the time-course of muscular adaptations in response to an exercise load would allow for comparisons to be made between exercises and hypothetical training volumes to be generated to inform exercise prescription. However, the inputs to these models include muscle forces (Wisdom et al., 2015), muscle activations (Zhou et al., 2017), and muscle-length (Zöllner et al., 2012), that cannot be calculated using purely experimental methods (e.g., inverse dynamics analysis). Therefore, MSK modelling is necessary to utilise these adaptation models and bridge the gap between computational biomechanics and practice.

There is an opportunity to understand MSK loading in hypogravity to inform astronaut exercise prescription. This is beneficial to both in flight exercise (i.e., actual hypogravity) and rehabilitation (i.e., emulated hypogravity) post-spaceflight. Therefore, the aim of this study was demonstrate the added value of a MSK modelling approach in informing exercise prescription in hypogravity with respect to purely experimental methods. The secondary aim was to describe the internal loading in hypogravity during plyometric hopping. The objective was to estimate muscle forces and joint reaction forces using MSK modelling and integrate them with a muscle-adaptation model.

## 2 Methods

**FIGURE 1:**
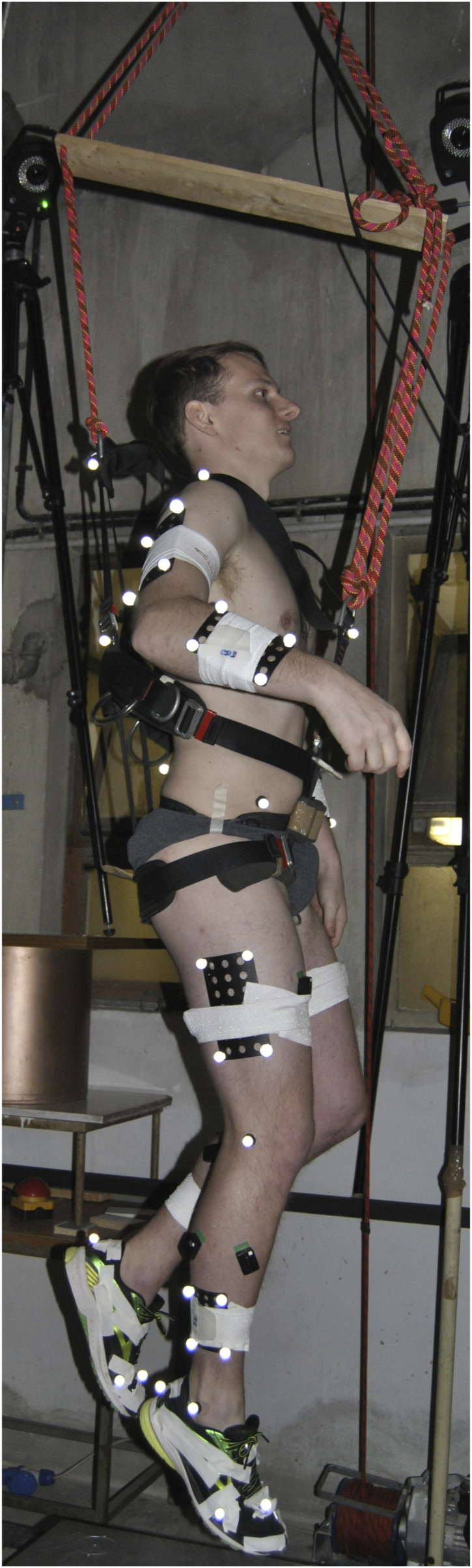
The participant performing single-legged hopping whilst attached the body-weight support system via a modified climbing harness. A full-body marker set was used to capture the motion of whole body during movement.

A case study of a single healthy and injury-free male (29 years, 1.82 m, 79.9 kg), who was healthy and performing single-leg vertical hopping was collected whilst attached to a body weight support system (BWSS). The study was approved by the Research Ethics Committee for Health (reference number EP 18/19,018) at the University of Bath. The participant was attached via a modified harness (Figure 1) under five emulated hypogravity conditions to capture a full spectrum of gravity levels from lunar to Earth gravity (0.17, 0.25, 0.37, 0.50, 1 g). The harness was removed for the terrestrial gravity (1 g) condition. A metronome was set at 2 *Hz* to assist the participant in maintaining a consistent hopping frequency across conditions. The BWSS constituted a series of elastic bungee cords originating on a manually adjusted electric winch, and inserting onto the participant’s harness. A ceiling-mounted pulley aligned the system above a single static force-platform (9287BA, Kistler Instruments Corporation, Switzerland). The line-of-action was in excess of 17 m to minimise vertical fluctuations in the body weight support force (Pavei et al., 2015). A full-body marker set (200 Hz, Vicon MX, Oxford Metrics, United Kingdom), GRFs (2000 Hz) and BWSS force via an in-series load-cell (2000 Hz, REP Transducers, TS 300 kg). Five hops were extracted per condition for analysis.

A custom MATLAB (version R2017b, MathWorks Inc., United States) script was written to format the data for the simulation framework. Trials were cropped to 0.15 s prior to right foot touchdown (hopping side) and following the consecutive right foot touchdown. Inverse kinematics was performed via the OpenSim-Matlab API (version 3.3, Delp et al., 2007) to obtain joint angles and translations from the marker trajectories. Inverse kinematic results, GRF, and, where applicable, body weight support forces from the load cell were filtered at 6 Hz with a low-pass second-order Butterworth filter. Inverse dynamics analysis was performed to obtain net joint moments and forces. Body weight support forces were applied vertically to the pelvis centre of mass of the model. Third-order B-splines were then used to resample the data, and to calculate velocities and accelerations of the inverse kinematics data. The resampled kinematics, net joint moments and GRF were used as experimental data within the simulation framework.

### 2.1 Simulation framework

#### 2.1.1 Musculoskeletal model

Skeletal motion was modelled as rigid body dynamics using Newtonian mechanics, with compliant Hunt-Crossley contacts to model the foot-ground interaction. A generic full-body MSK model was utilised in this framework (Lai et al., 2017). The model consisted of 23-segments–ground, pelvis, torso, and, bilaterally, femur, patella, tibia-fibula, talus, calcaneus, toe, humerus, radius, ulna and hand–and 37 degrees of freedom. The hip, knee, ankle, and subtalar DOF were actuated by 80 Hill-type MTU, and the pelvis, torso and upper body were driven by 23 idealised torque actuators. Idealised torques were described as a function of activation and maximum torque. The foot-ground interaction was modelled with six-spheres per foot–four attached to the calcaneus and two to the toe segments. Hunt-Crossley equations, modified to be twice continuously differentiable (Serrancolí et al., 2019), were used to calculate the forces at each of the six spheres as a function of ground penetration and penetration velocity.

Three-element Hill-type muscle model formulations were used in this framework (Zajac, 1989). Briefly, the MTU consisted of a contractile component, a passive elastic component parallel to the contractile component, and a series passive elastic component. Active (force-length and force-velocity) and passive (parallel and series) force generation were modelled via the dimensionless equations presented by De Groote et al. (2016). The dimensionless equations were described by five parameters: maximum isometric force, optimum fibre length, pennation angle at optimum fibre length, maximum shortening velocity, and tendon slack length. The model was scaled to the participant’s anthropometrics using OpenSim’s scaling tool (Delp et al., 2007). Maximum isometric forces were scaled according physiological cross-sectional area (Lieber and Fridén, 2000). Muscle volumes were estimated for the participant’s height and mass (Handsfield et al., 2014) with a specific tension of 60 N⋅cm^2^, as done previously (Rajagopal et al., 2016). Maximum shortening velocities were assumed to be ten times the optimal fibre lengths, and the tendon force-length curves had a gradient of 35 at 4% strain, as done in a similar simulation framework (Falisse et al., 2019b). Pennation angles at optimum fibre length from the unscaled model were retained. Muscle lengths, velocities, and moment arms were parameterised with polynomials defined as a function of joint positions and velocities, as done previously (Van den Bogert et al., 2013; Falisse et al., 2019b). Polynomial coefficients were determined by placing the scaled model in random positions within and exceeding the expected range of motion. Raasch’s activation model (Raasch et al., 1997) was used to model excitation-activation dynamics of the MTUs with modifications by De Groote et al. (2009).

#### 2.1.2 Optimal control problem

A data-tracking simulation framework was formulated as optimal control problems (OCP). A direct collocation method was then used to discretise the OCP into a non-linear programming program (NLP) to track experimental kinematics, net joint moments, and GRFs for a single hopping trial. The goal of the simulation was to minimise a cost function to estimate muscle activations for a given trial whilst optimising for a set of state, *x*, control, *u*, and parameter variables, *p*. The framework is designed to track the experimental data with zero pelvis residuals to elicit a dynamically consistent solution, before estimating the joint reaction forces (JRF) from the simulated muscle activations.

Foot-ground contact sphere stiffness and damping (constant across all spheres) and their 3D position were optimised as parameters within the OCP (p_*cm*_). The remaining parameters were kept constant according to previous work (Falisse et al., 2019b), with the sphere radii set to 0.02 m. The state (x) and control (u) variables were selected to allow efficient numerical formulation of the MSK system. The skeletal dynamics were described by the model’s kinematics. The state variables, *q* and $\dot{q}$ , which correspond to the positions and velocities of the 37 DOF, respectively, were controlled by their accelerations, $\ddot{q}$ . Muscle activations, *a*_*m*_, and normalised tendon forces, $\tilde{F_{t}}$ , were introduced to describe the states of each muscle model with their first time derivatives, ${\dot{a}}_{m}$ and $\dot{\tilde{F_{t}}}$ , introduced as control variables (De Groote et al., 2016). The states of idealised torque actuators were described by their activations, *a*_*τ*_, and controlled by their excitation, *e*_*τ*_. Control variables were introduced for the GRFs, *u*_*GRF*_, as done previously (Serrancolí et al., 2019). This improves the convergence rate as the foot-ground contact sphere forces are subject to large fluctuations for small adjustments to the skeletal dynamics. Reserve actuators were added to muscle-driven DOF as control variables, *τ*_*res*_, that describe the instantaneous moment being produced, to help convergence of the simulations.

##### 2.1.2.1 Cost function

The cost function (*J*, Eq. 1) was formulated with a muscle sharing criterion (Eq. 2), data tracking terms (Eq. 3) and control variable regularisation terms (Eq. 4). Each term was weighted (*w*_1–3_ = [0.01, 1, 10]) via manual tuning to elicit accurate tracking and physiologically realistic simulations, and were kept constant once calibrated. Increased weight was placed on net joint moment tracking to ensure muscle forces were recreating accurate joint moments, and thus to give valid JRF estimations. Less emphasis was placed on tracking the vertical pelvis position to allow the simulation to move the model vertically to supplement the GRF tracking. To prevent large changes in kinematics and muscle activations and tendon forces between time points, increased weight was placed on minimising their control variables (i.e., $\ddot{q}$ , ${\dot{a}}_{m}$ and $\dot{\tilde{F_{t}}}$ ).

$$J=J_{\mathrm{effort}}+J_{\mathrm{tracking}}+J_{\mathrm{DynCon}}$$ (1)

Muscle redundancy was solved by minimising the sum of muscle activations squared. Muscle activations were multiplied by their muscle volume expressed as a percentage, $P_{V_{j}}$ , of all muscles according to Handsfield and colleagues (2014) to penalise the use of larger muscles.

$$J_{\mathrm{effort}}=w_{2}\overset{80}{\underset{j=1}{\sum }}\int_{t0}^{tf}{\left(P_{V_{j}}\cdot a_{j}\right)}^{2}\;dt$$ (2)

Tracking terms were formulated to minimise the sum of squared error between the simulated (i.e., $\hat{q}$ , ${\hat{u}}_{\mathrm{GRF}}$ , and $\hat{\tau }$ ) and experimental data (i.e., *q*, *GRF*, and *τ*). Each term was scaled to maintain equal weight within the cost function despite the different orders of magnitude. Angular and translational coordinates were scaled by 2° (*s*_*rot*_) and 0.02 m (*s*_*tr*_), respectively. The scale factors for the net joint moments (*s*_*τ*_ = 28.6) and GRFs were then derived as the moment and force values required to perform 1 unit of work for the corresponding scale factor for the kinematics. The scale factors for the anteroposterior and mediolateral forces reduced by 9.81 to account for gravity to give the final scale factors (*s*_*GRF*_ = [5.1, 50, 5.1, 5.1, 50, 5.1]).

$$\begin{array}{ll} J_{\mathrm{tracking}}=\; & w_{2}\overset{34}{\underset{i=1}{\sum }}\int_{t0}^{tf}{\left(\frac{q_{i}-\hat{q_{i}}}{s_{\mathrm{rot}}}\right)}^{2}\;dt\\ & +\;w_{1}\overset{3}{\underset{i=1}{\sum }}\int_{t0}^{tf}{\left(\frac{q_{i,\;trans}-{\hat{q}}_{i,\;trans}}{s_{tr}}\right)}^{2}\;dt\\ & +\;w_{2}\overset{6}{\underset{n=1}{\sum }}\int_{t0}^{tf}{\left(\frac{GRF_{n}-{\hat{u}}_{GRF_{n}}}{s_{\mathrm{GRF}}}\right)}^{2}\;dt\\ & +\;w_{3}\overset{31}{\underset{i=1}{\sum }}\int_{t0}^{tf}{\left(\frac{\tau_{k}-\hat{\tau_{k}}}{s_{\tau }}\right)}^{2}\;dt \end{array}$$ (3)

Minimisation of control and reserve actuator variables was included to improve the dynamic consistency of the solutions, as done previously (Falisse et al., 2019b; Haralabidis et al., 2021). The control variables were scaled with their corresponding upper bounds (*s*_*Bound*_, see *Direct Collocation* below), whilst the *τ*_*res*_ were scaled with constant value identified during testing (*s*_*res*_ = 2).

$$\begin{array}{ll} J_{\mathrm{Control}}=\; & w_{2}\overset{37}{\underset{i=1}{\sum }}\int_{t0}^{tf}{\left(\frac{{\ddot{q}}_{i}}{s_{\mathrm{Bound}}}\right)}^{2}\;dt\\ & +\;w_{3}\overset{80}{\underset{j=1}{\sum }}\int_{t0}^{tf}{\left(\frac{{\dot{\tilde{F}}}_{t_{j}}}{s_{\mathrm{Bound}}}\right)}^{2}\;dt\\ & +\;w_{3}\overset{80}{\underset{j=1}{\sum }}\int_{t0}^{tf}{\left(\frac{{\dot{a}}_{m_{j}}}{s_{\mathrm{Bound}}}\right)}^{2}\;dt\\ & +\;w_{2}\overset{12}{\underset{m=1}{\sum }}\int_{t0}^{tf}{\left(\frac{\tau_{res_{j}}}{s_{\mathrm{res}}}\right)}^{2}\;dt \end{array}$$ (4)

##### 2.1.2.2 Direct collocation

**FIGURE 2:**
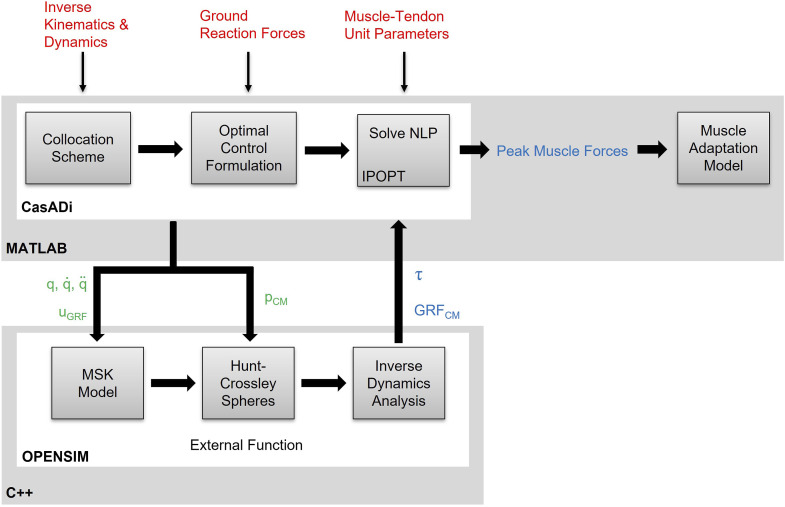
Schematic of the integrated direct collocation and OpenSim simulation pipeline. Experimental kinematics, joint net moments and muscle-tendon unit parameters are fed into the direct collocation pipeline written in MATLAB/CasADi. The OpenSim methods are formulated as an external function, written in C++ and embedded into a dynamic link library, and the generalised coordinates (*q*), velocities $\left(\dot{q}\right)$ , and accelerations $\left(\ddot{q}\right)$ , ground reaction force controls (*u*_*GRF*_) and contact model parameters (*p*_*CM*_) are passed as inputs to calculate ground reaction forces from the contact model (*GRF*_*CM*_) and joint net moments (*τ*) at each time step of the simulation. Optimised quadricep muscle forces were extracted and passed into the muscle adaptation model. Experimental data are highlighted in red, design variables in green, and variables derived from design variables in blue.

A direct collocation method was used to convert the OCP into a NLP using *Legendre-Gauss-Radau* quadrature. The framework was implemented in MATLAB (version R2017b, MathWorks Inc., United States) and CasADi (Andersson et al., 2019), and was solved using IPOPT (Wächter and Biegler, 2006). A modified OpenSim and SimBody release was utilised to allow for algorithmic differentiation of function derivatives (Falisse et al., 2019a). Net joint forces and moments via inverse dynamics, and Hunt-Crossley forces were estimated at each mesh point as a function of model kinematics (i.e., *q*, $\dot{q}$ , and $\ddot{q}$ ), external force (*u*_*GRF*_, and body weight support forces), and foot-ground contact model parameters (Figure 2).

Foot-ground contact model parameters were included as static parameters¸ *p* = [*p*_*CM*_]. The state $\left(x=\left[q,\dot{q},a_{m},{\tilde{F}}_{t},a_{\tau }\right]\right)$ and control $\left(u=\left[\ddot{q},{\dot{a}}_{m},{\dot{\tilde{F}}}_{t},u_{\mathrm{GRF}},e_{\tau }\right.\right)$ trajectories were discretised into 50 equally spaced mesh intervals (Ackermann and van den Bogert, 2010). State trajectories were parameterised with third-order polynomials via Four-points per interval. Design variables were scaled between −1 and 1 to improve the numerical conditioning of the NLPs (Betts, 2010).

Constraints were imposed to maintain system dynamics and physiologically realistic solutions. Dynamics of the state variables were enforced as implicit constraints, imposed at each collocation point, to ensure continuity of the equations of motion. The excitation-activation dynamics of the idealised torque actuators were formulated explicitly as linear first-order approximations given an electromechanical delay (35 *ms*) (Falisse et al., 2019b). Muscle-tendon unit polynomials were evaluated at each mesh point. Path constraints were then imposed to ensure physiological realism of the solution. These constraints were imposed to ensure consistency between the muscle moments and inverse dynamics, the idealised torques and inverse dynamics, the *u*_*GRF*_ and the foot-ground contact model forces, and the tendon and muscle forces (i.e., Hill-equilibirum). Raasch’s activation model was imposed on the muscle activations via two inequality constraints based on the activation (*t*_*a*_ = 0.015 s) and deactivation (*t*_*d*_ = 0.06 s) time constants (De Groote et al., 2009). An additional constraint enforced dynamical consistency by restricting pelvis residuals to be zero.

##### 2.1.2.3 Initial guess

Two initial guesses were formulated, and the solution with the lowest cost function value used in further analysis. A data-informed initial guess was generated by extracting experimental data for kinematics (i.e., *q*, $\dot{q}$ , and $\ddot{q}$ ) and GRFs. The remaining state and control variables were set to their corresponding lower bounds. A second guess was generated where all variables were set to zero, or, where the bounds did not cross zero, their corresponding lower bound. For both guesses, the contact sphere parameters were set to the same values. The stiffness and damping coefficients were set to 1e^6^ *N* ⋅ *m* ^−2^ and 2 s ⋅ *m* ^−1^, respectively. The same initial guesses as Falisse et al. (2019b) were used for the sphere positions.

### 2.2 Data analysis

Simulation performance was assessed by calculating maximum errors and root mean squared errors (RMSE) between experimental and simulated data. Acceptable tracking errors were determined based on recommendations by Hicks et al. (2015). Reserve actuators were considered acceptable if they did not contribute more than 5 *N* ⋅ *m* or 10% to overall net joint moments.

Sagittal plane lower-limb joint kinematics and kinetics were analysed for each trial, and mean (±SD) values calculated for each gravity level. Peak and the time-integral (i.e., the impulse) of the knee net joint moments were calculated as representative of traditional biomechanical analysis of joint loading. Angular impulse was included to account for differences in contact times, and to capture cumulative load. A joint reaction force analysis was performed to provide additional insight into mechanical load of the knee joint. The vertical force applied to the tibia by the femur was extracted to represent the compressive joint force. Data were normalised to the participants 1 g body weight (BW). Peak force and joint reaction impulse (the time integral of the JRF) was calculated to compare joint load per hopping cycle.

A muscle-adaption model was used to assess the feasibility of hypothetical training volumes on knee muscle homeostasis. Quadriceps muscle forces were extracted and input into a muscle adaption model (Eq. 5; Wisdom et al., 2015). Extracting internal muscle forces from the optimisation and combining them with additional information contained within the muscle adaptation model allows for the estimation of potential hypertrophic effects of the observed movement.

$$\dot{CSA}\left(t\right)=\frac{1}{\tau }{\left(\frac{CSA^{\max}-CSA}{CSA^{\max}-1}\right)}^{\delta }F-F^{0}$$ (5)

The adaptation model estimates the rate of change in cross-sectional area (*CSA*) of a muscle based on the degree of overload experienced, the muscle’s current cross-sectional area, a physiological maximum possible A (*CSA* ^max^), and a minimum load threshold required to trigger adaption (*F*_0_). The quadriceps force, *F*, was taken as the cumulative active and parallel passive forces from the Rectus Femoris, Vastus Lateralis, Vastus Intermedius, and Vastus Medialis muscles, normalised to their summed maximum isometric force parameters contained within the scaled OpenSim model. The physiological threshold, *F*_0_, was taken as 0.2 to replicate 20% one-repetition maximum. As a percentage of one-repetition maximum, this value has been shown to be sufficient to elicit hypertrophic benefits following resistance training under the right conditions (Schoenfeld, 2013). The baseline *CSA*, *CSA* ^max^, and parameters, *τ* and *δ*, that determined the model shape were determined based on data from DeFreitas et al. (2011). These data included thigh lean mass *CSA* determined via peripheral quantitative CT (pQCT) during a resistance-training intervention given to healthy, untrained, young adult men, which was considered appropriate for the case-study participant. DeFreitas and colleagues (2011) defined the minimal worthwhile increase in *A*, based on the calculations of Weir (2005), of 3.37% in their data. A hypothetical training volume was calculated by estimating the number of sets required to elicit this minimum worthwhile increase was by using the same target repetitions per set (12) and sessions per week (3) as DeFreitas et al., (2011).

## 3 Results

All 5 trials per gravity condition optimised for both initial guesses. Kinematics were tracked to within a maximum error of 3.8° and 0.08 m across all DoF and trials. After removing the pelvis vertical translation (0.08 m), which was allowed more flexibility in the optimisation, the maximum tracking error fell below 0.02 m. Maximum tracking error across all GRF components and net joint moments were within 0.08 BW and 0.81 N⋅m⋅kg^−1^, respectively.

**FIGURE 3:**
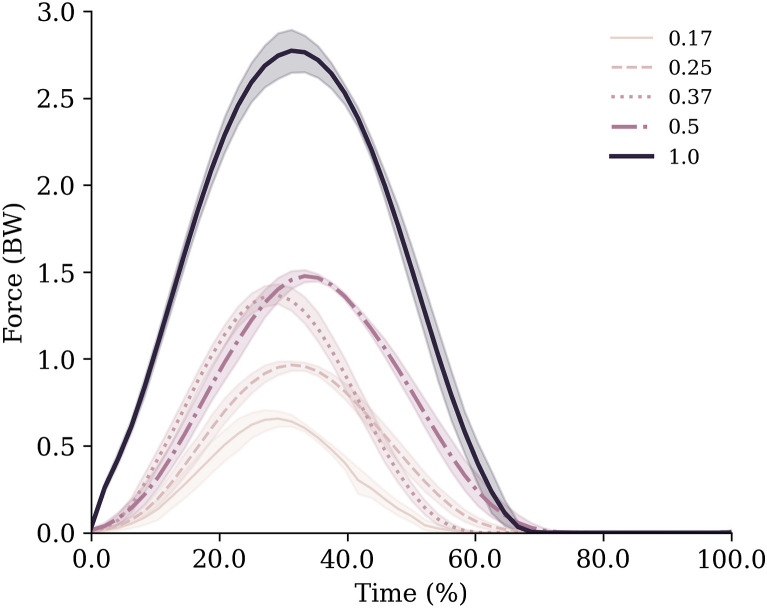
Vertical ground reaction forces normalised to body weight for each gravity condition. Data are mean (lines) and standard deviation (shaded area) across all trials.

Ground contact time remained relatively stable between 0.17–0.37 g (0.17 g = 0.29 ± 0.02 s; 0.25 g = 0.27 ± 0.02 s; 0.37 g = 0.28 ± 0.01 s), but increased with gravity thereafter (0.50 g = 0.32 ± 0.02 s; 1.0 g = 0.34 ± 0.01 s). This was partly due to variation in hopping frequencies, which were not consistent across gravities despite the metronome being used (0.17 g = 1.88 ± 0.08 Hz; 0.25 g = 2.33 ± 0.14 Hz; 0.37 g = 2.03 ± 0.04 Hz; 0.50 g = 2.16 ± 0.08 Hz; 1.0 g = 1.85 ± 0.01 Hz). Despite this, the monotonic increases in GRFs (Figure 3) with gravity were enough to ensure impulses increased as gravity in a similar manner (Table 1).

**TABLE 1:** Peak vertical ground reaction forces and impulses for one hopping cycle (contact and flight phases) at each gravity condition.

	Peak	Impulse
Ground Reaction Force		
0.17 g	0.67 ± 0.06	0.09 ± 0.01
0.25 g	0.97 ± 0.04	0.13 ± 0.01
0.37 g	1.37 ± 0.08	0.20 ± 0.01
0.50 g	1.48 ± 0.05	0.24 ± 0.01
1 g	2.76 ± 0.15	0.57 ± 0.00

Variables are in BW (forces) or BW⋅s (impulses).

**TABLE 2:** Peak net joint moments, joint reaction forces and their respective impulses for one hopping cycle (contact and flight phases) for each gravity condition.

	Hip		Knee		Ankle	
	Peak	Impulse	Peak	Impulse	Peak	Impulse
NJM						
0.17 g	−0.46 ± 0.09	2.10 ± 0.29	0.55 ± 0.07	2.49 ± 0.32	0.22 ± 0.02	1.05 ± 0.16
0.25 g	−0.61 ± 0.12	2.34 ± 0.36	0.55 ± 0.08	2.05 ± 0.28	0.34 ± 0.02	1.64 ± 0.17
0.37 g	−0.61 ± 0.09	2.75 ± 0.38	0.44 ± 0.12	1.70 ± 0.51	1.16 ± 0.13	5.57 ± 0.61
0.50 g	−0.62 ± 0.17	3.03 ± 0.80	0.56 ± 0.24	2.34 ± 1.06	1.46 ± 0.17	7.02 ± 0.82
1 g	0.70 ± 0.35	3.79 ± 1.89	3.16 ± 0.15	16.09 ± 0.97	3.56 ± 0.18	25.23 ± 0.61
JRF						
0.17 g	4.49 ± 0.59	1.18 ± 0.08	2.49 ± 0.18	0.76 ± 0.04	1.61 ± 0.08	0.36 ± 0.01
0.25 g	4.74 ± 0.58	1.03 ± 0.09	2.85 ± 0.22	0.68 ± 0.05	2.08 ± 0.14	0.39 ± 0.03
0.37 g	5.43 ± 0.37	1.27 ± 0.08	4.63 ± 0.23	1.01 ± 0.05	4.85 ± 0.38	0.80 ± 0.06
0.50 g	5.27 ± 0.68	1.28 ± 0.12	5.07 ± 0.37	1.13 ± 0.05	5.15 ± 0.45	0.89 ± 0.08
1 g	6.64 ± 0.58	1.83 ± 0.09	15.40 ± 2.87	2.99 ± 0.50	13.10 ± 0.83	3.02 ± 0.16

Net Joint Moment (NJM) variables are in N⋅m⋅kg^−1^ (moments) or N⋅m⋅kg^−1^⋅s (impulses), joint reaction force (JRF) variables are in BW (forces) or BW⋅s (impulses).

**FIGURE 4:**
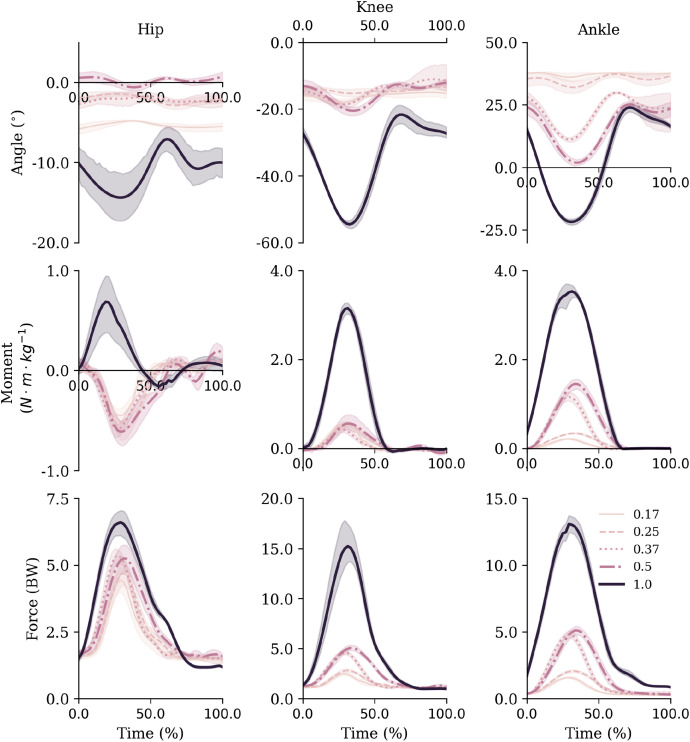
Sagittal plane joint angles (top row), net joint moments (middle row) and joint reaction forces (bottom row) for the hip, knee and ankle. Mean and standard deviations are represented via the lines and shaded areas, respectively. Note that the *y*-axis scales are not equivalent across joints to facilitate visibility of all gravity level curves over facilitating comparisons between joints. Extension and plantarflexion are defined as positive.

Inspection of the joint mechanics highlighted that in hypogravity the ankle was the main joint that adapted to the increased external demands (Figure 4). Specifically, the range of motion gradually increased at the ankle during ground contact, whilst the knee and hip remained relatively constant. There was also a decoupling of the knee and ankle net joint moments from the hip in hypogravity. The lower two joints acted to control flexion with extension moments throughout contact the lower-limb, whereas a net flexion moment was seen at the hip with a more consistent joint position. As gravity increased to 1 g the three joints all displayed a net extension moment and a substantially increased range of motion.

Similarly to the joint angles, peak moments and impulses were relatively consistent across hypogravities for the hip and knee joint, despite the increase in external demands. Whilst they monotonically increased at the ankle (Table 2). However, the increase in gravity between 0.50 and 1 g saw a substantial increase in peak net joint moments and net joint impulses.

Similar to the net joint moments, the influence of gravity on vertical joint reaction forces (vJRF) and impulses (vJRI) was different for hip than the knee and ankle joints (Figure 4). There was a clear increase in peak vJRF and vJRI with gravity at the knee and ankle, with an almost 3-fold increase between 0.50 and 1 g (Table 2). Whilst the vJRF at the hip did tend to increase with gravity, the standard deviations demonstrate that the increases were not meaningful - not even as gravity increased to 1 g.

**FIGURE 5:**
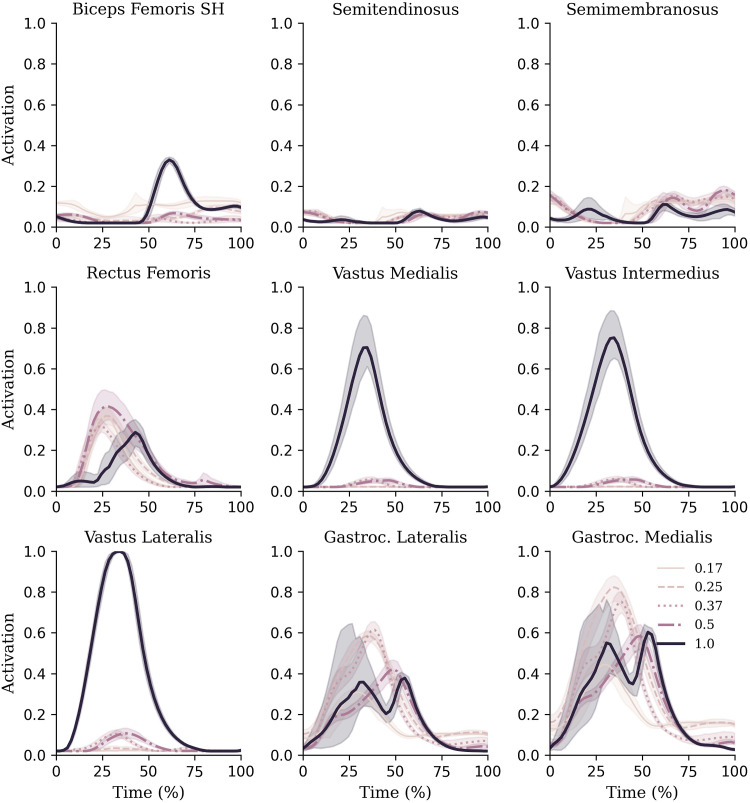
Muscle activations of knee and ankle muscles during each gravity condition. Mean and standard deviations are represented via lines and shaded areas, respectively.

**FIGURE 6:**
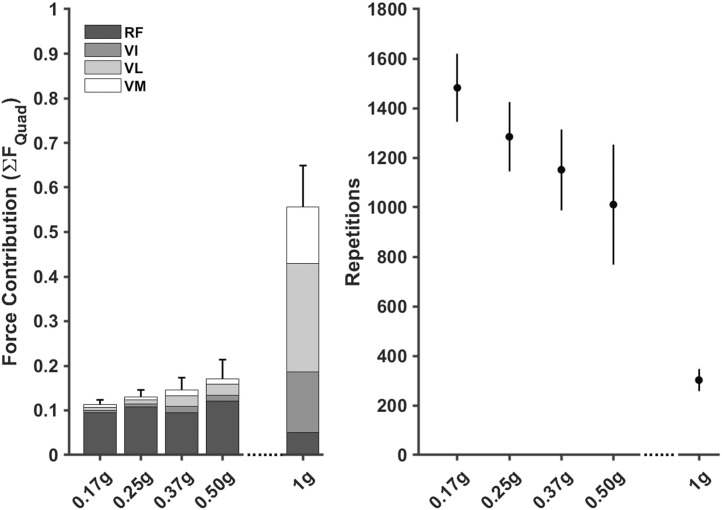
Normalised knee extensor muscle forces, stacked to represent the Quadricep muscle group, for each gravity condition (left) were used to estimate repetitions required (mean ±95% CI) to elicit a 3.37% increase in cross-sectional area (right). RF = Rectus Femoris, VI = Vastus Intermedialis, VL = Vastus Lateralis, VM = Vastus Medias.

There were two distinct patterns in how the muscle forces were distributed amongst the quadriceps between hypogravity (0.17–0.50 g) and terrestrial gravity (1 g) to achieve the observed increase as gravity increased (Figure 6). In hypogravity, the RF was the main contributor to quadriceps muscle force. However, at 1.0 g the Vasti muscles, particularly the VL, became more dominant, with the RF contributing less force at 1.0 g than at any of the other gravity levels. This is reflected in the muscle activations that shows the vasti muscles were activated less than 10% in hypogravity, but were fully active in 1 g (Figure 5).

When passed into the muscle adaptation model, the estimated repetitions required to elicit a 3.37% increase in muscle CSA decreased as gravity increased (0.17 g = 1,426 ± 99; 0.25 g = 1,334 ± 138; 0.37 g = 1,164 ± 130; 0.50 g = 1,025 ± 184; 1.00 g = 252 ± 14) (Figure 6).

Hypothetical training volumes demonstrated that the number of estimated repetitions decreased as gravity level increased. The model estimated that between 40–29 sets of 12 repetitions would need to be completed per session (i.e., three times per week) as gravity increased from 0.17 to 0.50 g to achieve a 3.37% increase in CSA. In contrast, only 7 sets would need to be completed three times per week at 1.0 g.

## 4 Discussion

In this study, we estimated the lower limb joint reaction forces during single-legged hopping at different levels of hypogravity. Such results were achieved by using a MSK modelling approach, which provided key information beyond that given via inverse dynamics analysis. Inverse dynamics analysis highlighted that the ankle joint was primarily used to support the increase in external load (from 0.17 to 0.50 g) in hypogravity. In contrast, at 1 g the external demand was met through a combination of increased net joint moments through the lower-limb. In isolation, this suggests that single-legged hopping in hypogravity should be used to target the ankle musculature, whereas in 1 g it should be considered a whole limb task. However, MSK modelling analysis showed that the quadriceps femoris muscle forces, particularly from the rectus femoris, increased monotonically with gravity and the joint reaction forces at the knee were comparable to the ankle at all gravity levels.

These findings give important insight into MSK loading in simulated hypogravity using body-weight support, highlighting that estimating internal loading (muscle and joint forces) are more appropriate for MSK load profiling.

It was observed that the ankle experienced the biggest change in net moment whilst hopping in hypogravity. Across the four hypogravity conditions, the ankle monotonically increased its range of motion and peak net plantarflexion moment. In contrast the hip and knee, although the absolute angle of the hip changed, they remained relatively static and provided consisted peak net flexion (hip) and net extensor (knee) moments. Akin with other jumping movements, the load experienced by the hopping limb was done with the objective of reversing the centre of mass trajectory. This was reflected in the GRFs, with large magnitudes and consequently greater impulses, required to overcome the increased gravitational demands. These results suggest that in hypogravity below 0.5 g, the MSK system prefers to utilise the ankle to meet the external demands of the task. Previous literature that compared inverse dynamics across hypogravities (either with parabolic flight or ground-based analogues) are not available, which meant other paradigms were considered to provide context to our findings. A recent systematic review of walking gait suggests that when body weight support is above 50% (i.e., ≤ 0.5 g) the range of motion and peak net joint moments decrease at all three lower-limb joints (Apte et al., 2018). This suggests that during gait the increased load is distributed along the limb, as opposed to focused on the ankle, in hypogravity. Drawing comparisons between gait and jumping/landing paradigms is difficult, and there is a sparsity of research of jumping biomechanics in hypogravity contexts. Previous research has been conducted on supramaximal and submaximal jumping, which is analogous to jumping in hypogravity where the external work demands are altered. Wade et al. (2018) added mass to participants via a weighted vest to increase external work demands to raise the centre of mass. In contrast to our results, all three joints demonstrated an increase in peak net joint moments with increased body mass. This suggests that when external demands are within the capabilities of the MSK system, we may prefer to utilise one joint to meet external demands rather than through a combination of all joints when demands are high.

One explanation of the ankle dominant moment generation is that utilising the ankle joint is an effective strategy to minimise energy usage due to favourable MSK properties. As summarised by Vanrenterghem et al. (2004), prioritising the ankle joint during submaximal jumping is beneficial for two reasons: 1) the reduced inertia and horizontal orientation of the foot segment, relative to more proximal segments, reduces the energy requirements of the movement, and 2) the long tendons in the distal limb (e.g., the Achilles) allow the contractile apparatus to contract at a slower velocity whilst storing elastic energy. This culminates in reduced active force generation relative to utilising all three lower-limb joints. Alternatively, previous work in a hypogravity context has suggested that since preoperative systems, such as the otholithic organs, are still under the influence of environmental gravity, participants make adjustments to their coordination to control landings from jumping tasks (Gambelli et al., 2016). Thus, the participant in our study may have simplified the coordination demands by preferentially selecting the ankle.

As gravity increased to 1 g there was a shift in the strategies used to meet the increase gravitational demand. The range of motion and peak net joint moments all increased in the hip, knee and ankle at 1 g, with greatest changes observed at the knee and hip. The knee underwent an almost 3-fold increase in range of motion and almost 6-fold increase in peak net extension moment, whilst the magnitude of net hip moment remained similar but shifted from net flexor to net extensor moment. This is in comparison to a more modest 1.3-fold increase in peak plantarflexor moment at the ankle. Of particular note, the hip shifted from a net flexion moment to a net extension moment between 0.5 g and 1 g, suggesting the load shifted from the flexors to the extensors in this interval. This may have been an artifact of the BWSS which will have been providing between 390 N (0.5 g) and 650 N (0.17 g) vertical force underneath the pelvis. In fact, it is possible that the BWSS force was working to move the pelvis into posterior tilt (i.e., extending the hip) meaning the net hip moment was flexor to maintain a neutral position. There is evidence in the literature that there is greater change in the knee and hip with increasing external demands. When BWSS changes between 50% and 0% (i.e. 0.5 g–1 g), greater increases in peak knee and hip net joint moments are typically observed during walking (Apte et al., 2018). When increasing jump height from submaximal to maximal, there is a greater step increase in joint range of motion and work done seen at the hip and knee joints than at the ankle (Lees et al., 2004; Vanrenterghem et al., 2004; Wade et al., 2018). This suggests that the ankle joint approaches maximum effort at lower intensities compared to the other two joint, which might suggest the need for greater gravities to target the hip and knee muscles and joint. Utilising lower hypogravities (≥0.5 g), either with gravity replacement systems in flight or with BWSS during rehabilitation, appears to be a necessary strategy to target the hip and knee joint structures during hopping exercises. Future work should include gravity levels between 0.5–1 g to gain a more complete picture of how the load on the lower-limb changes within this hypogravity interval.

Applying MSK modelling to these data provided new insights into the load experienced by the lower-limb beyond that gained from traditional biomechanical analyses. To demonstrate this, the results highlighted that the quadriceps muscle force increased monotonically to meet the increasing knee extension moment as gravity increased. This aligns with previous research that has shown net joint moments increase when walking with progressively less body weight support (i.e., increasing gravity) (Apte et al., 2018). The normal conclusion from this level of analysis would be that increasing gravity leads to increased loading on the knee musculature. However, being able to quantify the individual contributions of the quadriceps muscles highlighted this was not necessarily the case. In fact, not only did the Rectus Femoris shift from providing the majority of muscle force in hypogravity, it contributed less force at 1 g relative to all hypogravity conditions. This was initially an unexpected outcome, as it is logical to hypothesise that the 6-fold increase in gravitational forces would require more contribution from the Rectus Femoris, even if the increase across the quadricep muscles was not uniform as gravity increased. However, there is some evidence in the literature that might explain this phenomena. Previous research has shown that biarticular muscles work to transfer power between joints while the monoarticular muscles work to produce larger active forces (van Ingen Schenau and Bobbert, 1993; Jacobs et al., 1996). This is reflected in other research that has found a smaller contribution from the Rectus Femoris relative to Vasti muscles when walking (Sasaki and Neptune, 2006), running (Sasaki and Neptune, 2006; Hamner and Delp, 2013) and jumping (Pandy and Zajac, 1991) in 1 g. From our data, further analysis into the Rectus Femoris revealed a shift in RF muscle behaviour between 0.5 and 1 g. Specifically, the biarticular Rectus Femoris was being lengthened to a greater extent than the monoarticular Vasti, due to increased hip and knee flexion at 1 g, which moved the Rectus Femoris into the descending-limb of the force-length relationship. This compromises its ability to produce force, and therefore supports the notion that its role would be to transfer force down the leg. From a computational perspective, this made the Rectus Femoris more expensive in the cost function due to greater activation being required to achieve the same force output, thus encouraging the optimisation to prioritise the Vasti muscles to solve the problem. It is difficult to be certain which explanation led to the observed results, and it would require further work into the role of bi- and monoarticular muscles in hypogravity to determine. What this does demonstrate is MSK modelling approaches can provide additional information to analyse human movement in hypogravity and during body-weight support studies. This information would suggest that single-leg hopping at 1 g would better target the Vasti muscles than the Rectus Femoris. With the addition of more gravities and movements, it would be possible to compare between modalities to ensure specificity and overload of desired muscle groups.

Providing muscle forces alone would allow for progressive overload to be monitored, but it is difficult to transfer this to a training program, such as the number of sets and repetitions to complete. To help address this and to further demonstrate the added value of MSK modelling, the muscle adaptation model highlighted single-leg hopping in hypogravity would not be a feasible method to increase muscle CSA. It was predicted that single-leg hopping in hypogravity (0.17 g–0.50 g) would require more than 29 sets of 12 repetitions based on a training schedule of three times per week. This is less practical to achieve within a reasonable time-frame (e.g., a week) given the other operational requirements of the astronaut. In contrast, only 7 sets were estimated when hopping at 1 g, which may be more feasible to achieve in a realistic time-frame. For comparison, the participants from DeFreitas et al. (2011) achieved the minimal worthwhile increase (3.37%) between weeks 1 and 2; or, assuming their participants met the target volume, after 6–12 sets of leg exercises. However, we are not suggesting that this particular muscle-adaptation model accurately predicts the required training volume to mitigate against spaceflight induced muscle atrophy. The mechanisms of muscle hypertrophy are poorly understood and the model used here is based on the assumption that the muscle adapts in response to mechanical tension, and does not account for muscle damage and metabolic stress as mechanisms for hypertrophy (Schoenfeld, 2010). Expanding on this, an adaptative response is not fully determined by the volume of exercise completed but is a complex interaction between training volume, nutrition and recovery. Where the benefit of this approach currently lies is in the ability to make comparisons between exercise modalities. If a comprehensive repository of movements and gravity levels can be created for which normative loading profiles can be quantified for specific muscles and joints, exercises can be graded according to their potential clinical benefit. This approach has been employed previously allowing for exercise prescription to be personalised relative to patient injury and rehabilitation stage (Van Rossom et al., 2018). In a hypogravity context, this research effort is already underway (Herssens et al., 2022) and would allow training interventions to be assessed with further research (e.g., with randomised control trials), and for practitioners to use to supplement their expert knowledge when designing astronaut exercise programs. It is acknowledged that providing well validated tools is another barrier to overcome in the implementation of MSK modelling in practice (Fregly, 2021). However, as our understanding of mechanobiology and MSK adaptation to load improves, the validity of MSK adaptation models should improve in parallel.

It is important to recognise the limitations of this study. The case-study design prevents the generalisation of the MSK load magnitudes and muscle-adaptation model repetitions to the population-level. As per the study aims, it allowed for demonstration that MSK model is more appropriate for profiling MSK load than inverse dynamics, and for demonstrating trends in MSK load as gravity increases. It does not capture the variability in the MSK system between individuals. In practice, MSK load should be estimated on an individual-basis to gain insight into the MSK condition of the astronaut. Furthermore, a generic MSK modelling approach was adopted, whereby a generic model was scaled to the participant’s anthropometrics. It has previously been shown that subject-specific information, such as muscle-tendon unit parameters (Serrancolí et al., 2020), improves the estimation of muscle and joint forces. Given the case-study design, this does not influence this study as the results are not being generalised. However, this should be considered in future studies depending on their aim. A similar generic approach may be used for comparisons between exercise movements and gravities to identify the change in load between modalities, but subject-specific elements may be required for accurate estimation of load. Additionally, due to issues during data collection EMG data were not available to compare with and validate the optimised muscle activations. Given our aim was to develop a framework for future research, we do not believe this detracts from the key messages of this work. However, for future work to make comparisons between exercise paradigms it is recommended that validation steps are made, such as collecting EMG data, to inform exercise prescription. Another limitation is that the muscle adaption model does not capture the complexity of tissue remodelling. The model assumes the muscle can only increase in size, and does not capture the decrease in CSA that likely occurs when mechanical stimuli are removed (e.g., during sleep or spaceflight). That been said, the model’s parameters are calibrated against a 12-week training program study (DeFreitas et al., 2011), and will indirectly reflect the periods of rest within the shape of the relationship. While the absolute training volume should not be interpreted as a threshold for when a worthwhile level of hypertrophy will occur, comparisons can still be made between loading conditions to assess the feasibility of training volumes. Furthermore, body-weight support systems, such as that used in this study, are only able to replicate offset of mechanical load that occurs in hypogravity. While this aligns with the particular aims of this study, it is important to remember that actual hypogravity provides a physiological challenge to multiple systems of the human body (Vernikos et al., 2016). The information that the framework provides can be used to supplement other knowledge streams, such as previous experience, during exercise prescription, but should not be used in isolation due to the complex changes to physiology in hypogravity.

Exercise prescription, both during and following spaceflight, remains limited due to the presence of MSK adaptations following hypogravity exposure. Musculoskeletal modelling analyses were presented within this study as a valuable tool to estimate MSK loading at the level of the muscles and joints, which cannot be done via inverse dynamics analyses. From an inverse dynamics perspective, it was observed that in hypogravity the ankle was the main joint being loaded with net plantarflexor moment increasing monotonically with gravity during single-leg hopping. An observation that was not seen at the hip nor knee joint. However, muscle forces and joint reaction forces estimated using MSK modelling methods demonstrated that the knee musculature and joint were being increasingly loaded as gravity increased. This suggests that not only the ankle is being loaded during single-leg hopping, and with the additional of MSK modelling the load on the other structures can be accounted for. Furthermore, to help the implementation of MSK modelling methods to inform exercise prescription, a muscle-adaptation model was utilised to estimate hypothetical training volumes. This allowed for the volume and feasibility of the exercise to be considered when designing exercise programmes. Applying the approach outlined in this study to a repository of different exercise movements and gravity levels would provide a wealth of information to match to the programme goals and astronaut’s condition.

## Data Availability

The original contributions presented in the study are included in the article/Supplementary material, further inquiries can be directed to the corresponding author.
